# Touching and Hearing Unseen Objects: Multisensory Effects on Scene Recognition

**DOI:** 10.1177/2041669516664530

**Published:** 2016-08-23

**Authors:** Simon J. Hazenberg, Rob van Lier

**Affiliations:** Radboud University, Donders Institute for Brain, Cognition and Behaviour, Nijmegen, The Netherlands

**Keywords:** scene recognition, spatial updating, haptics, multisensory integration

## Abstract

In three experiments, we investigated the influence of object-specific sounds on haptic scene recognition without vision. Blindfolded participants had to recognize, through touch, spatial scenes comprising six objects that were placed on a round platform. Critically, in half of the trials, object-specific sounds were played when objects were touched (bimodal condition), while sounds were turned off in the other half of the trials (unimodal condition). After first exploring the scene, two objects were swapped and the task was to report, which of the objects swapped positions. In Experiment 1, geometrical objects and simple sounds were used, while in Experiment 2, the objects comprised toy animals that were matched with semantically compatible animal sounds. In Experiment 3, we replicated Experiment 1, but now a tactile-auditory object identification task preceded the experiment in which the participants learned to identify the objects based on tactile and auditory input. For each experiment, the results revealed a significant performance increase only after the switch from bimodal to unimodal. Thus, it appears that the release of bimodal identification, from audio-tactile to tactile-only produces a benefit that is not achieved when having the reversed order in which sound was added after having experience with haptic-only. We conclude that task-related factors other than mere bimodal identification cause the facilitation when switching from bimodal to unimodal conditions.

## Introduction

As we navigate through our environment, the retinal image is constantly changing; while some objects come into our field of view, others fade out. To keep track of these objects, we have to continuously update their positions in our surroundings with respect to our body, a process called spatial updating. Although the high spatial resolution that vision provides may be sufficient for such spatial tasks, events and objects often stimulate multiple senses at the same time. In fact, perception seems to be shaped by complex interactions between different sensory modalities (Eimer, 2004; Spence, 2007). When we are deprived of vision and we have to rely on the remaining senses such as hearing and touch, such interactions between intact senses may become even more relevant (Hötting & Röder, 2009). In the following experiments, we investigate whether haptic scene recognition can be influenced by object sounds that are played simultaneously with haptic exploration of the individual objects in the scene.

Several studies on spatial representations have found that recognition of spatial scenes depends on the position of the observer (Simon & Wang, 1998; Wang & Simons, 1999; Wraga, Creem-Regehr, & Proffitt, 2004). For example in the study of Simon and Wang (1998), participants had to learn a spatial scene comprising five familiar objects. Afterwards, the display was occluded from view and one of the objects was moved to another position. At the same time, the whole display rotated 47° or the display remained stationary and the observer walked to another viewpoint. Their results showed that scene rotations impair the detection of the moved object. This viewpoint dependency suggests that the scenes were represented with respect to the observer’s body, in an egocentric reference frame. In contrast to passive rotation of scenes, when observers actively moved to another viewpoint, the cost due to the difference in viewpoint disappeared. Apparently, movement-related vestibular and proprioceptive information can be used to automatically update spatial relations between objects and our bodies (Simons & Wang, 1998).

Although the above-mentioned studies have been performed in the visual domain, representations of space are not necessarily specific to one sensory modality. Rather, it has been suggested that input from different senses are unified to form spatial representations that are amodal in nature (Lacey, Campbell, & Sathian, 2007). Accordingly, several studies focused on the functional similarities between visually and haptically encoded spatial scenes (Giudice, Betty, & Loomis, 2011; Loomis Klatzky, McHugh, & Giudice, 2012; Newell, Woods, Menargh, & Bulthoff, 2005). For example, Newell et al. (2005) replicated the viewpoint dependency of visual scene recognition (Simons & Wang, 1998), but tested in addition blindfolded participants who had to explore scenes through touch alone. Like visual scene recognition, haptic scene recognition also depended on the viewpoint in which the scene was learned. Furthermore, subsequent studies showed that observer movement compensated for the cost due to viewpoint difference (Pasqualotto, Finucane, & Newell, 2005; Pasqualotto & Newell, 2007), indicating that representations of haptic spatial scenes can also be updated during movement.

Many studies investigated spatial performance within one modality at a time, but it is only in rare occasions that we have access to just one sensory modality. Indeed, input from different sensory modalities are often bound together in order to improve detection and localization of stimuli. There are many examples of such crossmodal interactions in which vision appears to guide hearing or touch (Eimer, 2004). For example, in the ventriloquist illusion, sounds are mislocalised toward a visual stimulus (Bertelson & Aschersleben, 1998). In contrast, others revealed that sounds can influence vision as well (Shams, Kamitani, & Shimojo, 2000) by showing that when a single flash is accompanied with multiple beeps, the number of perceived flashes increases. Extending this illusion to the tactile domain, it has been shown that the number of sounds affects the number of perceived touches as well (Hötting & Röder, 2004). As a final example of interactions between hearing and touch, in the parchment skin illusion, sounds affect the haptic perception of textured surfaces (Guest, Catmur, Lloyd, & Spence, 2002; Jousmäki & Hari, 1998).

Given the variety of studies showing interactions between different sensory modalities, it seems likely that spatial representations of scenes can be influenced by multisensory stimulation as well. Indeed, in a series of experiments, it has been found that recognition of haptic scenes benefits from seemingly task irrelevant visual input (Pasqualotto, Finucane, & Newell, 2013). This effect, however, depended on the moment that vision was available. That is, when participants were able to see their surroundings, but not the scene, during the initial trials of the experiments, performance improved during a subsequent block of trials in which participants were blindfolded. In contrast, when participants were blindfolded during the initial trials, no later benefit of visual information was found. It was argued that vision provides an environment-centered reference frame that facilitates a more enduring memory of the scene as compared with when only haptics were available. In a further study, Chan and Newell (2013) tested whether similar effects could be produced by having background auditory information instead of vision. In contrast, they found that background auditory information resulted in worse performance on a haptic scene recognition task.

Here, we investigated whether sounds may facilitate recognition of haptic scenes when sounds are object-specific and presented at the moment the objects are actually touched. To investigate this, we performed three experiments in which we employed an audio-tactile spatial recognition task. Like previous studies (Chan & Newell, 2013; Pasqualotto et al., 2005; Pasqualotto & Newell, 2007), participants were blindfolded and had to learn the scenes through touch. In one block of trials, object-specific sounds were played whenever objects were touched. In another block of trials, the sounds were turned off. Since both tactile information and sounds convey information about the identity of the objects, simultaneous audio-tactile stimulation may benefit spatial scene recognition. To check whether there is a differential influence of the order of presentation, we balanced block order between participants (i.e., from bimodal to unimodal and vice versa). In Experiment 1, we used geometrical objects and simple sounds. In Experiment 2, we used familiar objects (toy animals) and sounds that were semantically compatible with the objects (animal sounds). In Experiment 3, we replicated Experiment 1, but now participants had to explicitly learn to identify all objects and the specific object-sound couplings before performing the actual scene recognition task.

## Experiment 1

In this experiment, blindfolded participants learned through touch a scene comprising six geometrical objects. Then, two objects were swapped and the task was to report which of the objects changed position. Similar to previous studies (Simon & Wang, 1998), the scene either rotated or the scene remained stationary while participants walked to another viewpoint. To investigate the influence of sounds, in two audio-tactile blocks, sounds were played upon touching objects, while in two tactile-only blocks, sounds were turned off. Considering the results of Pasqualotto et al. (2013) in which multisensory (visual-tactile) facilitation depended on the timing of multisensory input, we tested the effect of different sequences as well.

### Methods

#### Participants

A total of 20 students from the Radboud University participated in this experiment (age 18 to 28; 16 females) for money or for course credits. Participants gave written informed consent and the study was approved by the local ethical committee.

#### Material

**Figure 1.**
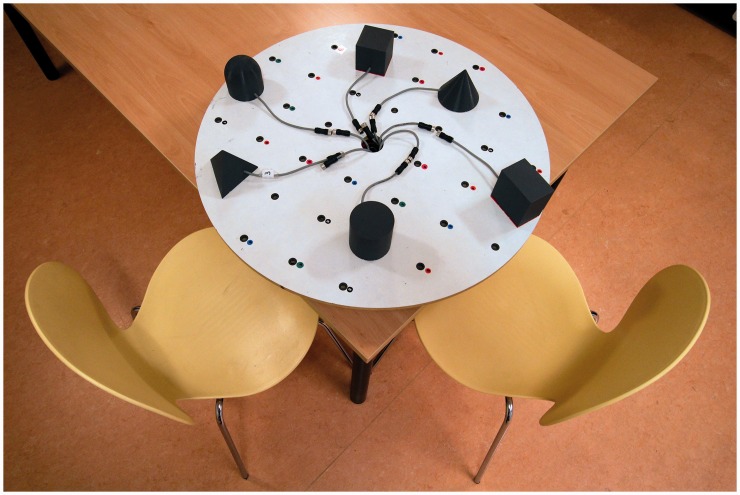
Set-up of Experiment 1. In each trial, the start position was the chair on the right. In the scene rotation condition, participants remained seated while the platform rotated 90° in counter clockwise direction. In the observer movement condition, participants walked to the other chair, while the platform remained stationary.

The stimulus set consisted of six objects which were placed in one of the 36 holes on a round platform (diameter = 70 cm). The holes were positioned in a grid-like fashion and were placed 10 cm apart from each other (see also Figure 1). The platform was positioned on a table with two chairs next to it.

Six geometrical objects of roughly the same size were created using a 3D printer. As can be seen in Figure 1, these include a cube, a rectangular cuboid, a cylinder, a pyramid, a cone, and half of a sphere. A small speaker was inserted into each object. Upon touching an object, this speaker played a sound that was specific to that object. The sounds were meaningless simple tones with each having a different frequency. Furthermore, for the rectangular cuboid, the cube, and the pyramid, the sounds comprised a series of short 300 ms tones with short 300 ms silent intervals in between the tones (having a frequency of 330 Hz, 420 Hz, and 520 Hz, respectively); for the cylinder, cone, and half a sphere, the sounds comprised continuous tones (having a frequency of 240 Hz, 295 Hz, and 380 Hz, respectively). Sounds were looped and continued playing until participants removed their hands from the object.

**Figure 2.**
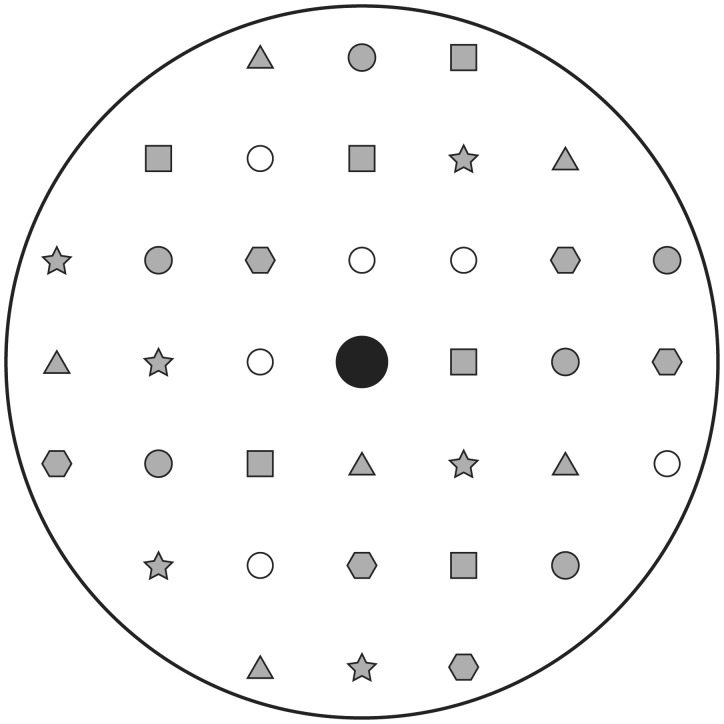
A schematic of the platform viewed from the top. The black disk in the middle represents the hole in the platform through which the cables run. The other smaller symbols depict the holes in which objects can be placed. Each of the five configurations that are used to create a scene is depicted by a gray symbol. The white disks are holes that were not used in the experiments.

To create the spatial scenes, all six objects were inserted into one of the holes in the platform. Rather than doing this randomly, five different configurations were specified with each having a different set of six holes (see Figure 2). This was done in order to minimize accidental grouping effects between objects (e.g., symmetry) that could facilitate the use of particular strategies. At the same time, for each configuration, the objects were distributed about equally across the whole platform. At the start of each trial, one of five configurations was randomly chosen. Then, each of the objects was randomly inserted in one of six holes making up the configuration. Thus, for each trial a different scene was created.

#### Procedure

The platform was placed on a table and the participants were seated in front of it so they could easily reach the objects. Participants were blindfolded at the start of the experiment. The experimental procedure comprised two phases, an exploration phase and a test phase. In the exploration phase, participants were given 45 s to explore and learn the spatial layout of the scene. They were instructed to use only their dominant hand. Next, the experiment leader swapped the positions of two randomly chosen objects. Care was taken that the noise of this action did not inform participants which objects changed position (e.g., by also lifting other objects and putting them back again; this took about 20 s). In the subsequent test phase, participants again explored the scene and had to report which of the objects changed position. There was no time limit during this phase, but when after 60 s no answer was given, the participants were prompted to do so.

Four experimental conditions were created based on a 2 by 2 design with rotation (scene rotation and observer movement) and sound (on and off). In the scene rotation condition, the platform rotated 90° in counterclockwise direction between the learning and test phase (a metal pin under the platform ensured that platform always rotated 90°). In the observer movement condition, the platform remained stationary while participants were required to walk to another chair that was positioned 90° in clockwise direction from the original chair. Furthermore, in two audio-tactile blocks, sounds were played when an object was touched during both exploration and test phase. In the remaining two tactile-only blocks, sounds were turned off during all trials.

The experiment was run in four blocks with each block consisting of trials from one condition. Additionally, the two audio-tactile blocks or the two tactile-only blocks followed each other. That is, one group of participants started with the two audio-tactile blocks and ended with the two tactile-only blocks. The other group of participants started with the two tactile-only blocks and ended with the two audio-tactile blocks. The presentation order of blocks was counterbalanced across participants. Each block consisted of 8 trials which resulted in a total of 32 trials. The experiment took about one and a half hours to complete.

Performance was recorded as proportion correct. When participants correctly chose both objects that switched position a 1 was scored; when only one object was correct, a 0.5 was scored, and when participants chose both objects wrongly, a 0 was scored.

### Results

Before analyzing the result, the proportions for each condition were averaged over trials. Next, to get normally distributed data, these proportions were transformed using an arcsine transformation. To investigate whether performance differed between conditions, we performed a repeated measures analysis of variance (ANOVA) on the transformed proportion correct with rotation (two levels: scene rotation and observer movement) and blocks (two levels: first two blocks or second two blocks) as within-subjects variables and sequence (start with audio-tactile blocks or start with tactile-only blocks) as between-subjects variable. A main effect of rotation was revealed, *F*(1, 18) = 14,269, *p* = 0.001, η^2 ^= 0.442, showing that scene recognition was better when participants moved to another viewpoint (mean = 1.052) as compared when the platform rotated (mean = 0.885).

**Figure 3.**
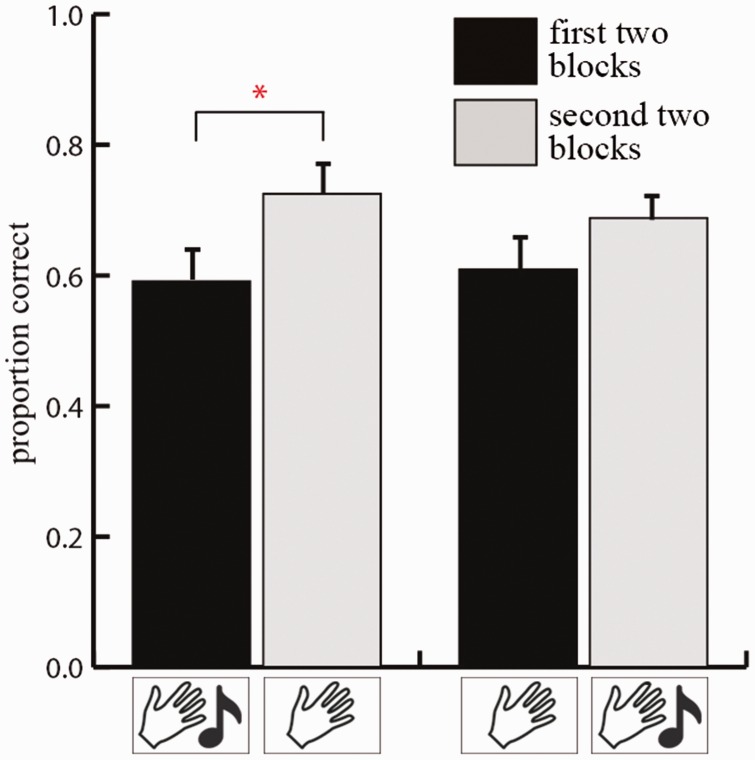
Results of Experiment 1. Performance in mean proportion correct for the first two blocks and the last two blocks. The left two bars depict the performance for participants who started with audio-tactile blocks and ended with tactile-only blocks and the right two bars depict the performance of participants who started with tactile-only blocks and ended with audio-tactile blocks. Error bars depict one standard error of the mean.

In addition, a main effect of blocks, *F*(1, 18) = 16.590, *p* = 0.001, η^2 ^= 0.480, showed that performance improved over time. That is, relative to the first two blocks (proportion = 0.902), performance was better during the second two blocks (proportion = 1.036). There was no main effect of sequence, *F*(1, 18) = 0.189, *p* = 0.669, nor was there an interaction between blocks and sequence, *F*(1, 18) = 1.673, *p* = 0.212. However, because a previous study showed that facilitation of multisensory input depended on the moment that this input was available (Pasqualotto et al., 2013), we ran separate *t*-tests for each group to investigate whether sounds could modulate this learning effect. As can be seen in Figure 3, the *t*-tests revealed that for participants who started with two audio-tactile blocks, performance improved during the following tactile-only blocks (proportion first two blocks = 0.898 vs. proportion second two blocks = 1.0745), *t*(9) = 4.000, *p* = 0.003. Running the same test for participants that started with two tactile-only blocks revealed no significant results, *t*(9) = 1.874, *p* = 0.094 (proportion first two blocks = 0.9057 vs. proportion last two blocks = 0.997).

None of the other interaction was found to be significant (rotation × sequence, *F*(1, 18) = 3.698, *p* = 0.070; rotation × blocks, *F*(1, 18) = 1.029, *p* = 0.393; rotation × sequence × blocks, *F*(1, 18) = 0.766, *p* = 0.393.

### Discussion

In this experiment, we investigated whether sounds contribute to haptic scene recognition. First, our results showed that when participants moved to another viewpoint, performance was better as compared with when the platform rotated. These findings replicate previous studies that use a similar task (Pasqualotto et al., 2005; Pasqualotto & Newell, 2007) and indicate that participants encoded the scene in an egocentric reference frame. This reference frame is not adequate during scene rotations, but it can be updated during observer movement (Simons & Wang, 1998). Second, our results reveal a general learning effect showing that scene recognition improved over time. However, it seems that the presence of sounds boosts learning as well. That is, the separate *t*-tests show that for participants for whom sounds were available during the first half of the experiment, performance improved during the last half of the experiment. For participants for whom no sounds were available during the first half of the experiment learning appears to be weaker. This suggests that learning depends, at least partly, on the availability of sounds during initial exploration of the scenes.

Note that during the first half of the experiment, participants of both groups performed about equally well on the task (compare first and third bar of Figure 3). A lack of an effect of sound at the start of the experiment can be explained by our choice of stimulus material. That is, because we used meaningless sounds, initially sounds did not provide information about the individual objects. Furthermore, although sounds may have been informative about the spatial location of objects, this appears not to be sufficient to improve scene recognition. The fact that improvement due to sounds emerge at a later time suggests that participants learn to associate the sounds with the individual objects. As a result, it may be that objects are recognized more easily at a later time, even when sounds are turned off. This may subsequently have facilitated memory of spatial scenes.

To explore this further, in the next experiment, we used familiar stimuli for which sounds and objects were semantically compatible. For these stimuli, associations between objects and sounds are rather intuitive and are likely to be more easily learned.

## Experiment 2

In this experiment, we investigated whether the learning effect due to sounds could be replicated using a different set of stimuli. Instead of using geometrical objects and abstract sounds, we now used familiar objects (e.g., toy animals) and combined them with semantically compatible sounds (e.g., vocalizations of animals). For example, touching the elephant triggered the sound of a trumpeting elephant. Previous studies showed that the recognition of pictures of animals is improved when matching vocalization of the animals are simultaneously presented (Molholm, Ritter, Javitt, & Foxe, 2004). It may be that vocalization of matching animals similarly facilitates haptic recognition of animals. Since both modalities provide convergent information about the identity of the objects, audio-tactile binding may be facilitated and this may be reflected in improved scene recognition.

### Methods

#### Participants

A total of 20 students (aged 18 to 29; 15 females) from the Radboud University participated in Experiment 2 for course credits or payment. These participants did not take part in any of the other experiments. Participants gave written informed consent and the study was approved by the local ethical committee.

#### Material

**Figure 4.**
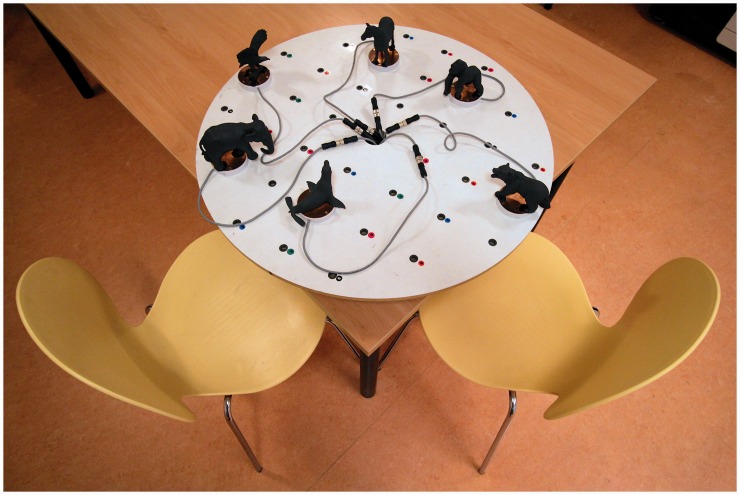
Set-up of Experiment 2.

The objects were toy figures comprising a set of distinctive animals. As can be seen in Figure 4, these included toy figures representing a horse, an elephant, a bear, a sea lion, a gorilla, and an eagle. The objects were roughly the same size and could easily be distinguished from each other. The sounds were semantically compatible with the objects and comprised a variation of animal calls. For example, the sound that was coupled with the bear consisted of three variations of the sound of a roaring bear (there was a short silent interval in between the roars). Like Experiment 1, the sounds were looped and continued playing until participants removed their hand from the object. Because of technical constraints, the sounds were played through headphones instead of through speakers that were fitted inside the objects. To do this, the objects were sprayed with electrical conductive paint so that to the moment of touching an object could be registered (signals were transposed to a computer to trigger the sounds). Apart from these changes, the procedure was exactly the same as Experiment 1.

### Results

**Figure 5.**
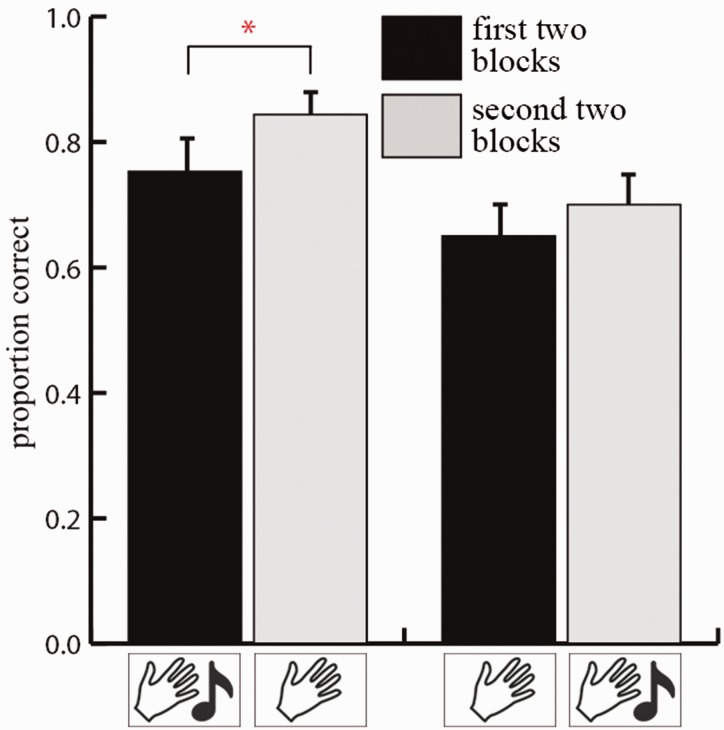
Results of Experiment 2. Performance in mean proportion correct for the first two blocks (black bars) and the last two blocks (light gray bars). The left two bars depict the performance for participants who started with audio-tactile blocks and ended with tactile-only blocks and the right two bars depict the performance for participants who started with tactile-only blocks and ended with audio-tactile blocks. Error bars depict one standard error of the mean.

To investigate whether performance differed between conditions, we performed a repeated measures ANOVA on the arcsine transformed proportion correct with rotation (two levels: scene rotation and observer movement) and blocks (two levels: first two blocks and second two blocks) as within-subjects variables and sequence (start with audio-tactile blocks or start with tactile-only blocks) as between-subjects variable. No main effect of rotation, *F*(1, 18) = 1.214, *p* = 0.285, was found. However, similar to Experiment 1, a main effect of blocks was revealed, *F*(1, 18) = 10.317, *p* = 0.005, η^2 ^= 0.364, showing that scene recognition was better during the last two blocks (proportion = 1.124) relative to the first two blocks (proportion = 1.032). Like Experiment 1, there was no main effect of sequence, *F*(1, 18) = 2.691, *p* = 0.118, nor was there an interaction between blocks and sequence, *F*(1, 18) = 0.600, *p* = 0.449. Importantly, however, running paired *t*-tests for each group separately revealed a similar pattern as was found in Experiment 1 with regard to the order of the conditions, audio-tactile versus tactile-only. As can be seen in Figure 5, when participants started with the audio-tactile blocks, performance improved during the following tactile-only blocks (proportion first two blocks = 1.098 vs. proportion last two blocks = 1.212), *t*(9) = 3.146, *p* = 0.012. In contrast, when participants started with the tactile-only blocks, no significant difference was found *t*(9) = 1.575, *p* = 0.150 (proportion first blocks = 0.967 vs. proportion last blocks = 1.037).

An interaction between blocks and rotation was also found, *F*(1, 18) = 5.798, *p* = 0.027, η^2 ^= 0.244. Paired *t*-tests revealed that performance improved only for the scene rotation condition, *t*(19) = 3.311, *p* = 0.004 (proportion first two blocks = 0.967 vs. proportion last two blocks = 1.119). For the observer movement condition, no significant results were found, *t*(19) = 1.103, *p* = 0.284 (proportion first two blocks = 1.098 vs. proportion last two blocks = 1.129). Furthermore, a marginal significant result, *t*(19) = 2.069, *p* = 0.052, reveals that during the first two blocks, participants are better in the observer movement condition as compared with the scene rotation condition. During the last two blocks, no significant differences between the observer movement condition and scene rotation condition were found, *t*(19) = 0.135, *p* = 0.894. No interaction between rotation and sequence was revealed, *F*(1, 18) = 1.189, *p* = 0.290, nor was there a three-way interaction between rotation, blocks, and sequence, *F*(1, 18) = 2.525, *p* = 0.129.

### Discussion

In this experiment, we set out to replicate the results of Experiment 1 and investigated whether the effect would lead to enhanced performance when semantically compatible audio-tactile stimuli are used. The results reveal an overall improvement that was similar to that of Experiment 1. Replicating the results of Experiment 1, the separate *t*-tests reveal significant improvement in scene recognition only for participants for whom sounds were available at the start of the experiment. In contrast, when sounds were not available during the initial trials, improvement in scene recognition appears to be weaker. At the same time, the results show that even when using highly familiar objects-sounds pairs (such as animals and their typical sounds), scene recognition did not immediately improve. Instead, benefits of sounds seem to emerge more indirectly by improving overall learning.

Contrary to previous reports (Pasqualotto et al., 2005; Pasqualotto & Newell, 2007) and the results of Experiment 1, we did not find overall improved performance when participants moved to another location as compared with when the scene rotated. It seems plausible that a possible facilitating effect of observer movement is simply canceled by the effect of familiarity with animals per se. That is, initial performance was already high, which was also reflected in the improvement for the more difficult scene rotation condition and not for the observer movement condition. Indeed, a benefit of observer movement relative to scene rotations was only found during the first two blocks, but not during the second two blocks. These results suggest that, although there may be an initial cost of scene rotations, participants quickly learn to recognize the familiar animals which then compensates for this cost.

Importantly, similar to Pasqualotto et al. (2013), visual tactile scene recognition task, we found a facilitating effect after the switch from bimodal to unimodal presentation. A difference is that in our experiment, the additional modality (sound) provided object identification information. In the next experiment, we focus on the role of bimodal object identification.

## Experiment 3

In the previous experiments, unimodal tactile scene recognition appears to benefit from the availability of sounds during the first half of the experiments. The similarity of this pattern for both experiments indicates that it does not seem to matter much whether or not the objects and sounds are semantically compatible (although overall scene recognition appears slightly better for Experiment 2). Thus, it appears that the switch from audio-tactile to tactile-only (rather then reversed sequence) is crucial to bring about an effect. It is still unclear, however, whether this is merely the result of an optimized implicit learning effect during the first blocks. That is, here we question whether this facilitating effect is to be attributed to increased object identification as such or whether the specific bimodal spatial setting is crucial. To investigate this, we replicated Experiment 1. However, before starting the experiment, participants had to explicitly learn to identify the objects and the specific object-sound couplings in a separate learning session up to a 100% identification score (both ways; see below). If we replicate previous results, then we may infer that it is not sufficient to learn the multisensory objects outside the context of the spatial task. That is to say that the multisensory stimulation within the spatial task setting is a crucial determinant of the switch asymmetry.

### Method

#### Participants

A total of 20 students (aged 18 to 28; 16 females) from the Radboud University participated in Experiment 3 for course credits or payment. These participants did not take part in the previous experiments. Participants gave written informed consent and the study was approved by the local ethical committee.

#### Materials

We used the same geometrical objects and sounds as were used in Experiment 1.

#### Procedure

The experiment started with an intensive learning session which was meant to learn all objects and sounds and the particular object sound couplings. To this end, participants were first allowed to freely explore the objects using their eyes, hands, and ears as long as they found necessary to learn which sound belongs to which object. They were told that after their exploration, two tests would follow to see whether the combinations were properly learned. In one test, an object was pointed out and the participant had to pick the correct sound out of three sounds. In the other test, a sound was played and participants had to pick the object that belonged to that sound. For both tasks, each sound and each object was tested twice. If mistakes were made, participants had to go back and learn the combination again. When no mistakes were made, the protocol was repeated while participants were blindfolded. After the learning session, the experiment started. The learning session took approximately 20 to 30 min. After the learning session, the actual experiment started. In this experiment, the same procedure was used as in Experiments 1 and 2.

### Results

**Figure 6.**
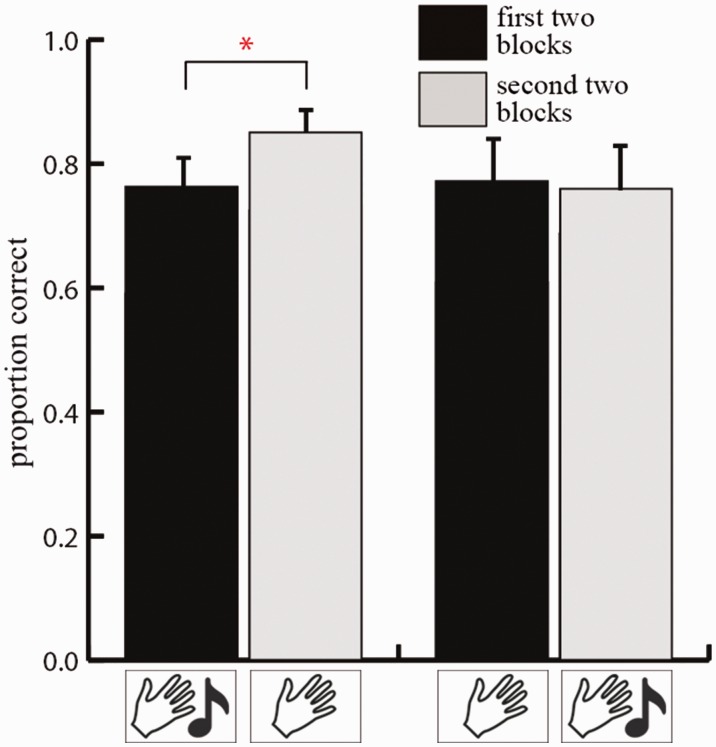
Results of Experiment 3. Performance in mean proportion correct for the first two blocks (black bars) and the last two blocks (light gray bars). The left two bars depict the performance for participants who started with audio-tactile blocks and ended with tactile-only blocks and the right two bars depict the performance for participants who started with tactile-only blocks and ended with audio-tactile blocks. Error bars depict one standard error of the mean.

To investigate whether learning the object–sound combination had an effect on the performance, we ran a repeated measures ANOVA on the arcsine transformed proportion correct responses with rotation (two levels) and block (two levels) as within-subjects variables and sequence as between-subjects variable (two groups). In contrast with the results of the previous experiments, no main effect of rotation, *F*(1, 18) = 2.917, *p* = 0.105, nor of block, *F*(1, 18) = 2.627, *p* = 0.209, was found. However, we did find an interaction between block and sequence, *F*(1, 18) = 5.966, *p* = 0.025, η^2 ^= 0.249. Figure 6 shows the mean proportion correct for the first two blocks and the second two blocks for each group. Paired *t*-tests revealed that the group that started with sounds improved on the task, *t*(9) = 2.915, *p* = 0.017, whereas the group that started without sounds performed about equally well over both halves of the experiment, *t*(9) = 0.573, *p* = 0.581. No interaction between rotation and sequence, *F*(1, 18) = 1.701, *p* = 0.209, nor between rotation and block, *F*(1, 18) = 0.054, *p* = 0.820, was found. The three-way interaction between rotation, block, and sequence was also not significant, *F*(1, 18) = 0.307, *p* = 0.587.

### Discussion

In this experiment, we used the same design, having the same shapes and sounds, as in Experiment 1. However, in contrast to previous experiments in which the shape of the objects and their sounds had to be learned on the go, here they were learned beforehand and outside the spatial context. The results largely replicated the results of Experiments 1 and 2 and again showed improved performance for participants that started with audio-tactile blocks, whereas scene recognition of participants that started with tactile-only blocks did not reveal such a switch advantage. We therefore reason that the differential order effect is not just driven by learning to identify objects but that learning within the specific multisensory spatial recognition task is a crucial aspect.

Interestingly, in contrast to the previous experiments, here we did not find a cost of scene rotation relative to walking to another viewpoint. In Experiment 1, there was a cost due scene rotation, whereas in Experiment 2, this cost disappeared during the course of the experiment. The results of Experiment 3 are in line with the idea that familiarity plays a modulating role. That is, explicitly learning to recognize the objects using auditory-tactile information apparently compensates for the cost of scene rotations, which fits with the notion that egocentric and allocentric frames of reference may exist in parallel and that the use of either of them depends on a variety of factors (e.g., Burgess, 2006).

## General discussion

In three experiments, we investigated whether sounds can contribute to haptic spatial scene recognition. In Experiment 1, we used geometrical objects and simple meaningless sounds. The results of Experiment 1 replicated previous findings (Pasqualotto et al., 2005; Pasqualotto & Newell, 2007) showing that haptic scene recognition can be updated during observer movement. More importantly, we showed that sounds did not immediately influence haptic scene recognition, but that the benefits emerge at a later stage. Specifically, the results show general improvement during later trials, and this effect appears to be enhanced by the availability of sounds during the first half of the experiment. Reversing this sequence diminished this effect, indicating that performance depended on initial context. In Experiment 2, we replicated these latter findings when familiar objects and semantically compatible sounds were used. This indicates that multisensory binding readily occurs and that it is not necessarily driven by semantic compatibility. As a cautionary note, although we replicated the effect, we did not find a significant interaction which, so far, suggests that the effect is rather modest. In Experiment 3, however, we again replicated the effect, but now found a significant interaction following a task in which objects-sound couplings were explicitly learned before the spatial scene recognition task. All in all, the results show that it is advantageous to be exposed to multisensory stimulation during the spatial scene recognition task itself.

Before discussing the results, note that there was a slight difference between experiments. That is, in Experiments 1 and 3, sounds originated from speakers that were inserted inside the objects while in Experiment 2, sounds were played through headphones. Although this could have affected the results, it seems unlikely. Previous studies, for example, showed that spatial accuracy of hearing is often guided by other sensory modalities (e.g., Bertelson & Aschersleben, 1998) and that integration of hearing and touch does not depend on colocation in space, but merely on co-occurrence in time (Murray et al., 2005). The results of Experiment 2 seem to be in line with this. Although sounds did not provide information about the location of objects, multisensory stimulation at the start of the experiment still resulted in improved scene recognition during later unimodal conditions. It appears that it is sufficient that audio-tactile stimuli are presented at the same time for binding to occur.

In any case, our results are partially in line with other reports on multisensory influences on spatial recognition. In particular, our results seem to parallel the findings of Pasqualotto et al. (2013), who found that benefits of task irrelevant vision on haptic scene recognition also depended on the moment vision was available. Specifically, seeing the surrounding room during initial trials improved performance at later trials. Improvement was not found when participants were blindfolded during initial trials. Explaining their results, they argued that seeing the room that the participants were in provided an environment-centered reference frame in which the haptic scene could be encoded. This would result in a viewpoint independent representation of the scene which contrasts with the use of a viewpoint depended representation when only haptic information was available. Interestingly, when instead of vision, background sounds were used, this differential order effect could not be replicated (Chan & Newell, 2013). It seems therefore unlikely that our results can be explained in a similar fashion (Pasqualotto et al., 2013), because in our experiments, sounds informed observers about the objects they touched and not so much about their surroundings. Thus, while the findings of Pasqualotto et al. (2013) and our results are consistent in showing a benefit when bimodal stimulation was presented first, the mechanisms underlying both effects might be different.

It should be noted further that audio-tactile couplings are less natural than visual tactile couplings. We mostly see (parts of) objects when touching them; hearing objects while touching them is rarer, although certain couplings are very natural indeed (e.g., scratching rough surfaces, or touching a cat that immediately starts purring). Furthermore, for sighted individuals, both the tactile and the auditory modality are not the primary modalities with regard to object localization and this may have been an important factor for the current results. That is, because the couplings in our experiment are more or less artificial (certainly so in Experiments 1 and 3), it is likely that more effort is needed to process multisensory stimulation (even if they are redundant) compared with unisensory stimulation. We suggest that the resources that are needed during audio-tactile conditions may persist after switching to unimodal conditions. In other words, it may be better to have all information (tactile and auditory) at once, even if they are redundant. Dropping the redundant auditory information leads to an improvement, whereas adding such information to initial tactile-only conditions does not have such an effect. It might very well be that having an additional modality combined with vision in a similar experimental set-up (e.g., visual-tactile versus vision-only) would not lead to the same differential order asymmetry—simply because vision is highly dominant with regard to identification and spatial processing.

Our findings are also consistent with studies showing that multisensory training can benefit performance during later unisensory tasks (Seitz, Kim, & Shams, 2006). Providing a possible account for such results, it has been argued that encoding under multisensory conditions activates a broad range of cortical networks. Compared with learning under unisensory conditions, this allows for the construction of richer representations. Furthermore, due to interconnectedness within the brain, such rich multisensory representations may later be accessed under unisensory conditions (Shams & Seitz, 2008). Related to this, future studies may investigate exactly when the availability of multisensory stimulation benefits scene recognition. For example, following the above, it seems likely that multisensory stimulation during exploration of the scene is sufficient for the benefit to occur, while multisensory stimulation during the test is not necessary.

The present results may have implications for applications that can be used by visually impaired humans. Since vision is the sense which is spatially the most accurate, it has been argued that visual experience is necessary for normal spatial perception to develop (Eimer, 2004). Pasqualotto and Newell (2007) investigated this by comparing recognition of scenes between sighted, late blind, and congenitally blind individuals. The results revealed similar performance between sighted and late blind participants. However, congenitally blind humans, who had no visual experience, performed significantly worse on the task. Furthermore, congenitally blind humans showed no sign of spatial updating when they walked to another viewpoint. In contrast, it has been shown that blind people may compensate for impairments by developing enhanced abilities in other modalities. For example, blind people sometimes show enhanced auditory skills as compared with sighted individuals (e.g., Röder et al., 1999). Although impaired auditory localization in blind people have been reported as well (Gori, Sandini, Martinoli, & Burr, 2013), it may be that simultaneous presentation of sound and haptics may benefit spatial skills of blind individuals.

Investigations on multisensory processing in spatial tasks have been mostly focused on audio-visual or visual-tactile couplings whereas studies on audio-tactile couplings have been relatively sparse. Here, we have shown that healthy participants are sensitive to audio-tactile couplings in a vision-deprived spatial scene recognition task. The couplings do not directly enhance localization, but induce enhanced performance in a subsequent tactile-only task—not the other way around.

## References

[bibr1-2041669516664530] Bertelson P. Aschersleben G. (1998) Automatic visual bias of perceived auditory location. Psychonomic Bulletin & Review 5: 482–489.

[bibr2-2041669516664530] Burgess N. (2006) Spatial memory: How egocentric and allocentric combine. Trends in Cognitive Sciences 10: 551–558. 17071127 10.1016/j.tics.2006.10.005

[bibr3-2041669516664530] Chan J. S. Newell F. N. (2013) The effect of non-informative spatial sounds on haptic scene recognition. International Journal of Autonomous and Adaptive Communication Systems 6: 342–365.

[bibr4-2041669516664530] Eimer M. (2004) Multisensory integration: How visual experience shapes spatial perception. Current Biology 14: R115–R117. 14986645

[bibr5-2041669516664530] Guest S. Catmur C. Lloyd D. Spence C. (2002) Audiotactile interactions in roughness perception. Experimental Brain Research 146: 161–171. 12195518 10.1007/s00221-002-1164-z

[bibr6-2041669516664530] Giudice N. A. Betty M. R. Loomis J. M. (2011) Functional equivalence of spatial images from touch and vision: Evidence from spatial updating in blind and sighted individuals. Journal of Experimental Psychology: Learning, Memory, and Cognition 37: 621–634. 10.1037/a0022331 PMC5507195 21299331

[bibr7-2041669516664530] Gori M. Sandini G. Martinoli C. Burr D. C. (2013) Impairment of auditory spatial localization in congenitally blind human subjects. Brain 137: 288–293. 24271326 10.1093/brain/awt311 PMC3891446

[bibr8-2041669516664530] Hötting K. Röder B. (2004) Hearing cheats touch, but less in congenitally blind than in sighted individuals. Psychological Science 15: 60–64. 14717833 10.1111/j.0963-7214.2004.01501010.x

[bibr9-2041669516664530] Hötting K. Röder B. (2009) Auditory and audio-tactile processing in congenitally blind humans. Hearing Research 258: 165–174. 19651199 10.1016/j.heares.2009.07.012

[bibr10-2041669516664530] Jousmäki V. Hari R. (1998) Parchment-skin illusion: Sound-biased touch. Current Biology 8: 190. 10.1016/s0960-9822(98)70120-4 9512426

[bibr11-2041669516664530] Lacey S. Campbell C. Sathian K. (2007) Vision and touch: Multiple or multisensory representations of objects. Perception 36: 1513–1521. 18265834 10.1068/p5850

[bibr12-2041669516664530] Loomis J. M. Klatzky R. L. McHugh B. Giudice N. A. (2012) Spatial working memory for locations specified by vision and audition: Testing the amodality hypothesis. Attention, Perception & Psychophysics 74: 1260–1267. 10.3758/s13414-012-0311-2 PMC3482114 22552825

[bibr13-2041669516664530] Molholm S. Ritter W. Javitt D. C. Foxe J. J. (2004) Multisensory visual-auditory object recognition in humans: A high-density electrical mapping study. Cerebral Cortex 14: 452–465. 15028649 10.1093/cercor/bhh007

[bibr14-2041669516664530] Murray M. M. Molholm S. Michel C. M. Heslenfels D. J. Ritter W. Javitt D. C. Foxe J. J. (2005) Grabbing your ear: Rapid auditory-somatosensory multisensory interactions in low-level sensory cortices are not constrained by stimulus alignment. Cerebral Cortex 15: 963–974. 15537674 10.1093/cercor/bhh197

[bibr15-2041669516664530] Newell F. N. Woods A. T. Menargh M. Bulthoff H. H. (2005) Visual, haptic and crossmodal recognition of scenes. Experimental Brain Research 161: 233–242. 15490135 10.1007/s00221-004-2067-y

[bibr16-2041669516664530] Pasqualotto A. Finucane C. M. Newell F. N. (2005) Visual and haptic representations of scenes are updated with observer movement. Experimental Brain Research 166: 481–488. 16034564 10.1007/s00221-005-2388-5

[bibr17-2041669516664530] Pasqualotto A. Newell F. N. (2007) The role of visual experience on the representation and updating of novel haptic scenes. Brain and Cognition 65: 184–194. 17845829 10.1016/j.bandc.2007.07.009

[bibr18-2041669516664530] Pasqualotto A. Finucane C. M. Newell F. N. (2013) Ambient visual information confers a contex-specific, long-term benefit on memory for haptic scenes. Cognition 128: 363–379. 23764999 10.1016/j.cognition.2013.04.011

[bibr19-2041669516664530] Röder B. Teder-Sälejärvi W. Sterr A. Rösler F. Hillyard S. A. Neville H. J. (1999) Improved auditory spatial tuning in blind humans. Nature 400: 162–166. 10408442 10.1038/22106

[bibr20-2041669516664530] Seitz A. R. Kim R. Shams L. (2006) Sound facilitates visual learning. Current Biology 16: 1422–1427. 16860741 10.1016/j.cub.2006.05.048

[bibr21-2041669516664530] Shams L. Kamitani K. Shimojo S. (2000) What you see is what you hear. Nature 408: 188. 10.1038/35048669 11130706

[bibr22-2041669516664530] Shams L. Seitz R. (2008) Benefits of multisensory learning. Trends in Cognitive Sciences 12: 411–417. 18805039 10.1016/j.tics.2008.07.006

[bibr23-2041669516664530] Simon D. J. Wang R. F. (1998) Perceiving real-world viewpoint changes. Psychological Science 9: 315–320.

[bibr24-2041669516664530] Spence C. (2007) Audiovisual multisensory integration. Acoustical Science and Technology 28: 61–70.

[bibr25-2041669516664530] Wang R. F. Simons D. J. (1999) Active and passive scene recognition across views. Cognition 70: 191–210. 10349763 10.1016/s0010-0277(99)00012-8

[bibr26-2041669516664530] Wraga M. Creem-Regehr S. H. Proffitt D. R. (2004) Spatial updating of virtual displays during self- and display rotation. Memory & Cognition 32: 399–415. 15285124 10.3758/bf03195834

